# Impacts of the SARS-CoV-2 pandemic on the dietary practices of university students in Germany

**DOI:** 10.3389/fnut.2024.1302308

**Published:** 2024-03-08

**Authors:** Jana O. Dreyer, Alexander C. Brandt, Silke Lichtenstein, Christian Sina, Martin Smollich

**Affiliations:** ^1^Dr. Rainer Wild-Stiftung, Heidelberg, Germany; ^2^Institute of Nutritional Medicine, University Hospital Schleswig-Holstein, Lüebeck, Germany

**Keywords:** students, SARS-CoV-2, dietary practices, sustainability, university catering, public health nutrition

## Abstract

**Purpose:**

The dietary practices (DPs) of university students are influenced by many external factors. Therefore, we investigate how the DPs of students in Germany changed during the SARS-CoV-2 pandemic, what the main motivations were for those changes, and what effect the closure of university catering had on the DPs of students.

**Methods:**

A total of 560 students from two universities in Lübeck (Germany) were surveyed online during a pilot phase. The final online questionnaire was subsequently administered at 10 other German universities (399 respondents). The questionnaire surveyed sociodemographic factors, dietary habits, food consumption frequencies, and the relevance of university catering before and during the SARS-CoV-2 pandemic.

**Results:**

Regarding changes in DPs, similarities to previous studies were found, especially positive eating behaviors and an increasing interest in health- and nutrition-related sustainability. Students prepared meals freshly more often during the pandemic; consumed legumes, plant-based meats and dairy alternatives more often; and reduced their consumption of meat and milk compared to before the pandemic. The consumption frequency of sweets also decreased. It was observed that students consider eating communal in the university canteen to be highly relevant for their social interactions, which was only possible to a limited extent during the pandemic.

**Conclusion:**

In Germany, the DPs of university students as well as criteria regarding health and sustainability changed during the first 2 years of the SARS-CoV-2 pandemic. The social aspect of DPs became evident due to closed university catering. Still, changes in dietary patterns and eating habits were positively related to health and revealed some differences in the cross section of the population.

## Introduction

Students in Germany, as everywhere else, faced new life conditions during the first weeks of the SARS-CoV-2 pandemic in 2020. Closed universities, online lectures, and restrictions on contact, travel, and business in the retail sector and in catering, including university catering, interrupted the daily routines of most university students, thus directly affecting established dietary practices (DPs) (1) DPs describes nutrition as a daily practice that includes more than actions limited to food preparation and intake (2). They are based on routines acquired over a lifetime, with frequent repetition of certain procedures (2). Thus, when routines have proven relevant and reasonable in the individual lifestyle, actions such as shopping or cooking are no longer reflected and are only led by the compressed, accumulated knowledge (3). Since the survey for this study comprises self-reported dietary habits and nutrition instead of a controlled survey protocol, the term DP is used to discuss the results of the survey appropriately (2).

The lives of university students are mainly structured around lectures, scientific research, and part-time jobs, which secure a living but especially influence DPs. These essential activities were disrupted by SARS-CoV-2 measures (4–7). Daily routines and social communities changed drastically, also affecting housing situations and household budgets (8). Students had to adapt their familiar DPs regarding shopping, cooking, and eating routines. Alternatives had to be found for out-of-home catering, especially for affordable meals from universities’ catering canteens, which were closed during the lockdown (9), playing a further important role in the communication and socializing of young adults (1). Many students live in two places (their hometown and their place of study) and often in shared flats; hence, they have on average more social contacts in more diverse environments than most other collectives (10–12). Regarding typical life and daily routines, the DPs of students were expected to be significantly affected by lockdowns with contact restrictions or the implementation of online lectures (1).

Initial studies described the effects of the SARS-CoV-2 pandemic on the DPs of the German population already in March 2020 (13–19).

Most studies described a distinct awareness of quality in the context of nutrition, including the preference to prepare fresh food, as well as the increasing relevance of aspects such as animal welfare, climate and species protection, labor conditions in the food sector, and the reduction of food waste (8, 14, 19). As zoonoses such as COVID-19 can be related to the food system, the sensibility according dietary and ecological sustainability arose (20–22). Several studies demonstrated that a sensibility occurred already in the early stages of the pandemic (7, 8, 22–28). One review concluded that the “new normal” seemed to imply a favorable development from the perspectives of nutrition physiology and social economics (29). Since crises usually induce a revision, and sometimes even innovation, of social norms, they will also affect the individual values and attitudes of students (30).

Several studies have addressed the dietary habits and nutrition of students (31–33). However, due to the methodologies and samples, the validity of the results is limited, especially when comparing students’ DPs pre and post the pandemic (12). The Göttingen studies by Busch et al. (18) found a decreased consumption of meat and dairy products in the first months of the pandemic, as well as an increase in the consumption of plant-based alternatives (18, 19). Students were also found to prepare their meals themselves; to try new recipes more often; and to consume fruits, vegetables, and sweets or cakes more frequently (34). However, sustainability-related DPs can be expected from most students, especially young females (2, 35). Regarding meat consumption and a vegan or vegetarian diet, these DPs relate to people with higher education (36–38). The use of university catering has been addressed by only a few studies, which state its relevance especially for young students (11, 39).

The purpose of the study was to investigate the extent to which the DPs of students may have changed in the context of the SARS-CoV-2 pandemic compared to the pre-pandemic period and during the pandemic. In our study, we investigate three questions:

1. How did the DPs of students in Germany change during the SARS-CoV-2 pandemic from 2020 to 2022?
2. What have been the main motivations for changes in DPs?
3. What was the effect of closed university catering on the DPs of students?

## Materials and methods

### Sample and survey

**FIGURE 1:**
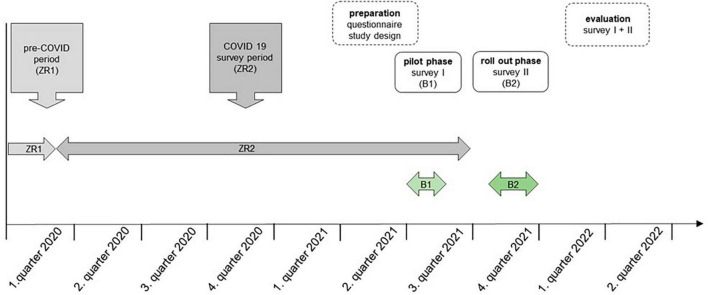
Survey period.

The study focused on the health- and sustainability-based nutritional practices of students at the beginning of 2020 and changes in those practices during the SARS-CoV-2 pandemic from 2020 to 2022 (Figure 1). The questions of the survey were developed by the authors on the basis of already published studies (18, 19) and supplemented by aspects that should also cover the question (e.g., university catering). The already published study designs served as a theoretical foundation and to ensure validity (18, 19). The questions were drafted in German. The survey was initially conducted on students from the University of Luebeck and the Technical University of Applied Sciences Luebeck (THL) in June and July 2021 (B1). In the winter term 2021/22, the survey was rolled out to students from other German universities (B2), ending on December 31 (Figure 1). For this phase, universities in Germany that offer study programmes on the subject of nutrition were consulted. The contact addresses of the universities were researched by the authors. The internet link to the website was distributed by the Dr. Rainer Wild-Stiftung and directly sent to participating universities. The universities then informed the students about the study by e-mail and provided the link to the survey at the same time. The sample size was not determined in advance and resulted from the number of questionnaires answered by the students during the pilot and rollout phase.

### Pandemic background

In 2021, the general mood in society regarding the SARS-CoV-2 pandemic in Germany was characterized by high stress and fatigue (1). After an initial decrease in COVID incidences in summer 2020, significantly more infections were reported after the holiday season and with colder weather at the beginning of the winter semester 2020/21 (19), resulting in a partial lockdown in November 2020. In the following months, vaccines became available, accompanied by discussions about safety measurements, vaccination campaigns, and compulsory vaccinations. This situation did not change before the first survey in summer 2021 (B1) (19). The second survey period was set at the end of 2021, when COVID incidences rose again.

### Online tool

Survey Monkey^®^ is a web-based survey service, was employed in this study. After the questions were established and the style of each question was chosen, the link to the questionnaire was exported and distributed via e-mail. Survey Monkey saves the IP address of each participant in order to prevent multiple participation. This excludes participants who have already taken part in the survey.. The results of the survey were exported as.csv files and analyzed using Microsoft Excel.

### Questionnaire

The online survey, with a total of 26 questions, focused on the living situation, income, and dietary habits of students. Food-related questions followed the concept of a food frequency questionnaire, which asks about the frequency of consumption based on a list of previously defined foods. Relevant changes were investigated by addressing two time periods with one question: before (ZR1: ≤ February 2020, Figure 1) and during the COVID-19 pandemic (ZR2: March 2020 until July 2021, Figure 1). Questions were single- and multiple-choice (limited to choosing two response options) with the additional option “no statement.” The questionnaire was in German language. The questionnaire was first designed in a pilot phase at two universities in Lübeck (Figure 1). The data analysis in the pilot phase ensured that the questions were easy to understand, and that the questionnaire covered the research question and was therefore valid for the roll out phase (Figure 1). The criteria of the CHERRIES guideline relevant to the questionnaire are fulfilled (40): The legal framework for data privacy and data storage of both institutes was listed on the start page of the study. It was also stated here how long the survey would take (15 min) and that the survey could be cancelled at any time. It was described that participants agree to these aspects by taking part. To get from the start page to the survey, participants had to click on a continue button. In addition, both institutes were named as investigators on the start page, as well as the options for contacting them and a contact person. The survey was voluntary, and no incentives were offered. The questions were always displayed in the same order and were therefore not randomized. Before completing the survey, participants were able to check and change their responses (back button).

### Data evaluation

The answers of all participants who did not complete the questionnaire in full were eliminated from the results. This was the only exclusion criteria for this study. The remaining data were analyzed collectively and in four separate sets: gender-dependent, income-dependent, depending on the living situation, and depending on the German federal state. Answers to questions with an optional field for input (e.g., study major, diet forms) were counted and evaluated to prevent mistakes due to typing errors. The mean and median were calculated for all counts of answers based on the represented number of participants. To determine significant changes, *t*-tests and Chi^2^-tests were conducted at a 95% significance level. The average frequency in days per week was predicted based on Equation 1:


(1) $$f_{a\,v}\,\left[\frac{d}{w\,k}\right]=$$

ne⁢v⁢e⁢r⁢y⁢d⁢a⁢y7*+n5⁢t⁢o⁢65.5*+n3⁢t⁢o⁢43.5*+n1⁢t⁢o⁢21.5*+n<10.5*nt⁢o⁢t⁢a⁢l

Comparisons between categories were always done with percentages, not with *n*-values. Ratios of female to male participants were calculated by dividing the percentages of the analyzed categories. A ratio of 1 reflected an even distribution, while values smaller than 1 represented a dominance of male participants, and values higher than 1 represented a dominance of female participants.

## Results

### Characteristics of the study sample

**FIGURE 2:**
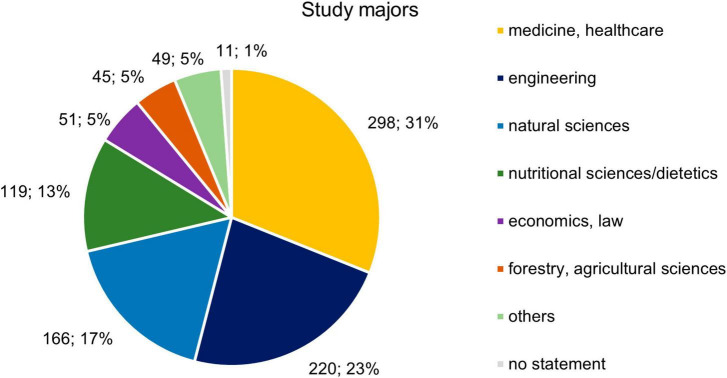
Distribution of students across different study majors (*n* = 959).

In total, 1,187 participants (female: 864 [73%]; male: 303 [26%]) started the online survey, and 959 of them (female: 705 [73.5%]; male: 239 [24.9%]) completed it. The participants studied mainly at four different universities: the majority studied at the University of Luebeck (39.3%), followed by the Technical University of Applied Sciences Luebeck (19%), the Hochschule Anhalt (18.8%), and the Friedrich-Schiller-University of Jena (17.1%). Furthermore, 31.1% of the participants studied medicine or other healthcare-related fields, followed by engineering (22.7%), natural sciences (17.3%), and nutritional sciences/dietetics (12.4%) (Figure 2). In terms of age, 67.8% of the participants were between 18 and 24 years old. The respondents’ housing situation was almost evenly distributed among the four types of housing (living alone: 20.6%, living with parents: 25.9%, living in a shared flat: 26.6, and living together with partner: 26.3%) (Table 1).

**TABLE 1:** Sociodemographic data of the study sample.

	Total, 100% (%) *n* = 959	Female (%) *n* = 705	Male (%) *n* = 239	Living in East-G (%) *n* = 342	Living in West-G (%) *n* = 615	Living in shared flats (%) *n* = 255	Living with partner (%) *n* = 252	Living with parents (%) *n* = 248	Living alone (%) *n* = 198
**Gender**									
Female	73.5	–	–	74.9	72.7	72.9	75.0	73.4	72.2
Male	24.9	–	–	23.1	26.0	24.7	22.2	26.2	27.3
**Age categories**									
18–24	67.8	71.8	56.5	74.9	63.9	72.9	45.6	87.1	66.2
25–29	23.3	20.6	31.4	16.1	27.3	23.9	35.7	10.1	22.7
30–39	7.0	5.4	11.3	7.3	6.8	2.7	14.3	2.4	8.6
≥40	1.6	1.8	0.4	1.5	1.5	0.0	4.4	0.0	2.0
**Study semester**									
1	11.1	11.8	9.2	23.4	4.2	5.9	6.7	22.2	9.6
2	12.6	13.2	11.7	2.0	18.5	11.4	14.3	13.7	10.6
3	10.8	12.3	5.9	23.4	3.9	11.8	9.5	11.7	10.6
4	16.3	16.3	15.9	1.2	24.7	16.9	17.1	14.1	17.2
5	9.4	8.7	10.9	21.3	2.8	11.0	6.3	10.1	9.6
6	14.2	14.3	14.6	2.3	20.8	14.5	18.3	9.7	14.1
7	7.4	7.9	6.3	11.7	4.9	7.5	6.7	8.5	6.6
8	6.7	5.4	9.6	1.8	9.4	5.5	8.7	4.4	8.6
9	3.0	3.1	2.9	4.7	2.1	5.1	1.6	1.2	4.5
10	3.3	2.6	5.4	0.9	4.7	5.9	2.4	1.2	4.0
>10	4.1	3.4	5.9	6.1	2.9	3.5	7.1	2.4	3.0
**Housing situation**									
Shared flat	26.6	26.4	26.4	27.2	26.3	–	–	–	–
With partner	26.3	26.8	23.4	20.2	29.8	–	–	–	–
With parents	25.9	25.8	27.2	31.3	22.8	–	–	–	–
Alone	20.6	20.3	22.6	20.8	20.5	–	–	–	–
**Having children**									
Yes	2.9	3.1	2.5	2.6	3.1	0.0	8.7	0.8	2.0
No	96.8	96.6	97.1	96.8	96.7	100.0	90.5	99.2	98.0
**Community size**									
≥100,000	57.1	58.4	52.3	43.0	65.0	74.1	64.7	23.4	68.7
20,000–99,999	18.6	17.2	23.0	27.8	13.5	18.8	14.7	18.5	22.7
5,000–19,999	8.6	8.5	9.2	9.4	8.1	2.4	8.7	17.3	5.6
<5,000	15.0	15.3	14.6	19.0	12.7	2.7	11.5	40.7	3.0
**Parents with university degree**									
Both parents	29.0	28.7	30.1	26.9	30.2	36.5	23.8	23.8	32.3
One parent	26.9	27.9	23.4	26.0	27.3	31.8	28.2	24.2	22.7
No parent	42.1	41.8	43.5	45.3	40.3	29.8	46.4	49.2	44.4

### Food expenses

In terms of food costs, 51.5% of respondents stated that they had had “no change” in food expenditure. Higher expenses of 10% up to 49% were reported by 35% of the respondents. Lower costs of 10% up to 49% were reported by 6.4% of the respondents (Table 2).

**TABLE 2:** Changes in food expenses from ZR1 to ZR2.

	Total (%) *n* = 959	Female (%) *n* = 705	Male (%) *n* = 239	f/m ratio	East-G (%) *n* = 342	West-G (%) *n* = 615	E/W ratio
Much lower costs (≥50% less)	1.7	1.7	1.3	1.36	1.5	1.8	0.82
Lower costs (10% to 49% less)	6.4	6.0	7.9	0.75	3.8	7.8	0.49
No change (± 10%)	51.5	50.9	53.6	0.95	53.2	50.6	1.05
Higher costs (10% to 49% more)	35.0	36.0	31.8	1.13	35.1	35.0	1.00
Much higher costs (≥50% more)	1.6	2.0	0.4	4.75	1.8	1.5	1.20
No statement	3.9	3.4	5.0	0.68	4.7	3.4	1.37

### Dietary habits

Regarding nutrition habits, 82.5% of the participants stated that they ate a “healthy and balanced” diet (Table 3). Furthermore, 50.8% of the participants indicated that their dietary habits had changed during the pandemic, with no significant differences between men and women. A more frequent consumption of functional foods or food supplements at ZR2 was confirmed by 12.8% of the participants (female: 14.2%, male: 9.2%).

**TABLE 3:** Statements about nutrition and eating habits.

Statement	Total (%) *n* = 959	Female (%) *n* = 705	Male (%) *n* = 239	f/m ratio
**My nutrition is healthy and balanced**				
Completely true	24.6	26.2	20.1	1.31
True	57.9	58.9	54.8	1.07
Not true	15.6	13.6	21.8	0.63*
Not true at all	1.9	1.3	3.3	0.38*
**My eating habits changed during the COVID-19 pandemic**				
Completely true	13.6	13.3	14.6	0.91
True	37.2	37.2	36.4	1.02
Not true	36.3	37.4	33.9	1.10
Not true at all	12.9	12.1	15.1	0.80
**I used food supplements, functional foods, etc., more often during the lockdown**				
Completely true	2.7	3.1	1.7	1.86
True	10.1	11.1	7.5	1.47
Not true	25.1	25.8	23.8	1.08
Not true at all	62.0	60.0	66.9	0.90

The * symbol means that this result is significant.

### Inspiration for dietary routines

When asked about their inspiration for dietary routines, 54% of the respondents stated that they found ideas through “self-motivation,” followed by “social media” (47.2%) (Table 4). Shopping (26.8%), family (26.5%), and friends (22.9%) were of almost equal importance for the students. Almost no student watched television to gather dietary inspiration (0.3%).

**TABLE 4:** Inspiration for nutrition (up to two answers per person) answers in italic have been given in the category “other.”

	Total (%) *n* = 959	Female (%) *n* = 705	Male (%) *n* = 239	f/m ratio
Self-motivation	54.0	51.1	62.3	0.82*
Social media	47.2	53.5	30.1	1.78*
During shopping	26.8	25.2	31.4	0.80
Friends	26.5	25.5	29.3	0.87
Family	22.9	22.0	25.5	0.86
*Cooking websites or apps*	3.3	3.0	4.2	0.71
*Books or magazines*	3.0	3.5	1.7	2.12
*Study*	1.1	1.3	0.8	1.53
*N\nutrition consultation*	0.4	0.3	0.8	0.34
*Television*	0.3	0.1	0.4	0.34
*Cafeteria*	0.1	0.1	0.0	–
*Doctor*	0.1	0.1	0.0	–
*Thermomix*	0.1	0.1	0.0	–
No statement	0.8	1.0	0.4	2.37

The * symbol means that this result is significant.

### Forms of diet

A vegetarian diet was the predominant form of nutrition (33.7% at ZR1) (Table 5). Both vegetarian and vegan diets became more popular during the lockdown. At ZR2, the share of students following a vegetarian diet rose to 38.4%, and a significant increase in veganism was also found (Table 5).

**TABLE 5:** Common diet forms at ZR1 and ZR2, by gender and new students (up to two answers per person).

	ZR1 (%) *n* = 959	ZR2 (%) *n* = 959	Difference (% pts)	f/m ratio (ZR2)	Sem. 1–3 ZR1 (%) *n* = 331	Sem. 1–3 ZR2 (%) *n* = 331
Vegan	14.4	20.9	6.5*	1.99*	14.8	20.8
Vegetarian	33,7	38.4	4.7	1.45*	29.6	39.3
Low carb, high protein	9.1	9.1	0.0	0.70	9.4	10.3
Intermittent fasting	12.3	14.5	2.2	1.46	13.9	15.7
*None*	9.0	8.6	−0.4	0.69	6.9	6.0
*Flexitarian*	4.4	4.6	0.2	1.55	3.6	3.3
Other	5.7	5.8	0.1	0.76	6.0	5.4
No statement	27.9	21.0	−6.9*	0.57*	30.8	21.1

f/m ratio, female/male ratio; Sem., semester. Answers in italics were given in the category “other”; all answers <3% have been summed up in “other.” Individual answers in the category “other”: Almased; anti-inflammatory; balanced; count calories; diet; ERNA Teller; Ernährungspyramide; high carb; intuitive nutrition; fast food; FODMAP; juice fasting; keto; lactose free; low meat; mixed diet; Mediterranean; pescetarian; plant based; halal; histamine-, lactose-, and sodium benzoate intolerance; low carb high fat; sugar free; Western diet. The * symbol means that this result is significant.

**FIGURE 3:**
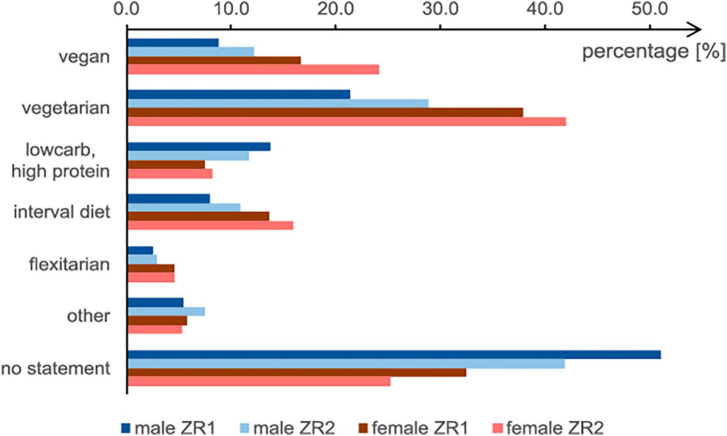
Gender differences in diet forms at ZR1 and ZR2.

The diet forms “low carb,” “high protein,” and “other” were dominant among male students. Significantly more female students followed a vegan or vegetarian diet at both ZR1 and ZR2 (Table 5 and Figure 3). Both men and women reported increases in vegetarianism and veganism. However, at ZR2, female students reported a greater increase in the vegan diet than male students did in the vegetarian diet.

**FIGURE 4:**
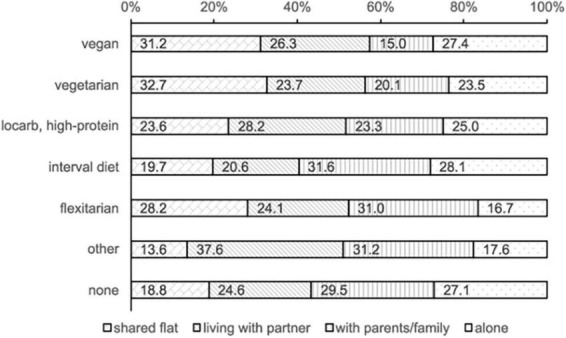
Special diets at ZR2 depending on student’s household type.

The number of vegetarian and vegan students differed among the four forms of housing (Figure 4). At ZR2, 32.7% of vegetarians and 31.2% of vegans were living in shared flats. The smallest proportions were students living with their parents (20.1 and 15%, respectively). Special diets, noted in “other,” were mainly followed by students living with their partner (37.6%).

### Food preparation methods and eating out of home

**FIGURE 5:**
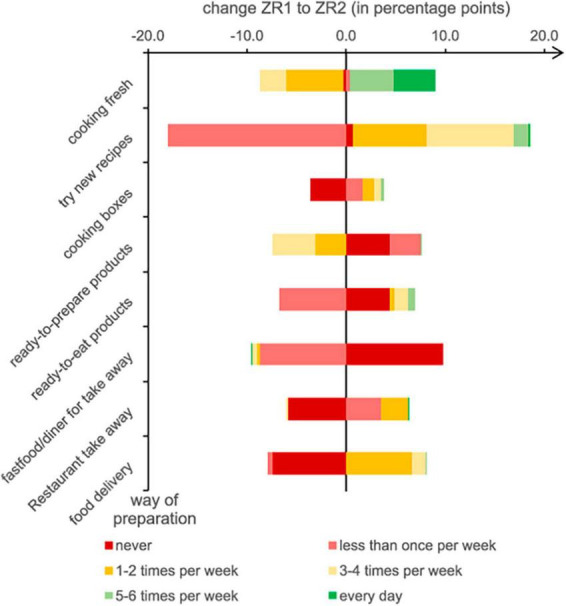
Changes in periodicity of food preparation from ZR1 to ZR2.

At ZR1, respondents were preparing fresh meals, on average, 3.96 days per week and using ready-to-prepare products 1.72 days per week. Ready-to-eat products (0.88 d/wk) and meals out of home were consumed even less frequently (Table 6). Significantly more respondents stated that they prepared fresh meals every day at ZR2 (22.8% at ZR2 vs. 18.7% at ZR1) and tried new recipes more frequently (1.49 d/wk at ZR2 vs. 1.07 d/wk at ZR1). This trend was especially evident among female students. In total, respondents reported consuming ready-to-prepare products less often (1.54 d/wk at ZR2 vs. 1.72 d/wk at ZR1) and ready-to-eat products more often (0.92 d/wk at ZR2 vs. 0.88 d/wk at ZR1). Takeaway services in restaurants (0.34 d/wk at ZR2 vs. 0.28 d/wk at ZR1) and delivery services (0.49 d/wk at ZR2 vs. 0.34 d/wk at ZR1) were also chosen more often. Female students reported preparing fresh meals more often than male students (4.23 d/wk among females vs. 3.37 d/wk among males) at ZR2. By contrast, men ate ready-to-eat products more often (1.14 vs. 0.76 d/wk) and more frequently bought food in diners or fast-food restaurants (0.81 d/wk at vs. 0.55 d/wk) (Figure 5).

**TABLE 6:** Frequencies of food preparation methods (ZR1 and ZR2).

	Daily use ZR1 (%) *n* = 959	Daily use ZR2 (%) *n* = 959	ZR1 (d/wk) *n* = 959	ZR2 (d/wk) *n* = 959	Difference (d/wk)	Change (%)
Fresh cooking	18.7	22.8	3.96	4.30	0.34	+8.9*
Try new recipes	1.0	1.2	1.07	1.49	0.42	+39.5*
Cooking boxes	0.0	0.0	0.04	0.11	0.07	+152.3*
Ready-to-prepare products	0.2	0.2	1.72	1.54	−0.19	−10.1
Ready-to-eat products	0.0	0.0	0.88	0.92	0.05	+7.2
Fast food/diner for takeaway	0.1	0.0	0.62	0.56	−0.07	−11.9
Restaurant takeaway	0.0	0.1	0.28	0.34	0.07	+20.9*
Food delivery	0.0	0.0	0.34	0.49	0.15	+45.4*

The * symbol means that this result is significant.

The greatest changes from ZR1 to ZR2 were observed for “try new recipes,” where the number of participants trying new recipes less than once per week decreased significantly (17.9%), while numbers in all other groups increased. The same pattern, albeit less pronounced, was observed for ready-to-eat products. Fewer participants used ready-to-prepare products or fast food/diner meals. Increases were greatest for cooking fresh, food delivery, and cooking boxes.

Bars with negative values indicate a decrease in the number of participants choosing the related category. The wider the range, the greater the changes.

### Fruits and vegetables

At ZR1, vegetables were consumed on 5.41 days per week, slightly more frequently than fruits (4.73 d/wk). The daily intake of vegetables and fruits was confirmed by 48% and 40.3% of participants, respectively. These proportions increased at ZR2 to 50.6% for vegetables and 43.3% for fruits (Table 7). Moreover, 57.7% of all respondents ate vegetables or fruits every day. A significant increase was found for the consumption of legumes, with the mean consumption frequency increasing by 7.8%, from 2.25 d/wk at ZR1 to 2.42 d/wk at ZR2 (Table 7).

**TABLE 7:** Consumption frequencies of different food products at ZR1 and ZR2.

Product	ZR1 (d/wk) *n* = 959	ZR2 (d/wk) *n* = 959	Difference (d/wk)	Change (%)	Female ZR2 (d/wk) *n* = 705	Male ZR2 (d/wk) *n* = 239
Vegetables	5.41	5.49	0.08	+1.5	5.76	4.71
Fruits	4.73	4.79	0.06	+1.3	5.13	3.80
Legumes	2.25	2.42	0.18	+7.8*	2.47	2.28
Meat, sausages	2.03	1.58	−0.45	−22.3	1.24	2.57
Fish	0.61	0.59	−0.02	−3.0	0.52	0.76
Meat substitute	0.85	1.22	0.37	+43.0*	1.28	1.08
Milk products	3.40	3.00	−0.41	−11.9	2.98	3.03
Cheese	3.30	2.98	−0.32	−9.8	2.94	3.14
Milk substitutes	2.18	2.76	0.58	+26.4*	3.10	1.74
Chocolate, sweets	3.01	3.00	−0.01	−0.3	3.20	2.36
Salty snacks	1.32	1.39	0.07	+5.7	1.42	1.29
Cereals, etc.	2.99	2.95	−0.04	−1.3	3.14	2.41
Cornflakes, etc.	0.93	0.87	−0.06	−7.0	0.82	1.01
Soft drinks	0.92	0.92	0.00	+0.1	0.77	1.30
Sugar-free soft drinks	0.62	0.68	0.06	+9.3	0.64	0.77
Alcoholic beverages	0.98	1.01	0.03	+2.8	0.93	1.23
Energy drinks	0.28	0.31	0.02	+7.3	0.23	0.51
Specialty coffees	0.76	0.77	0.01	+1.0	0.89	0.42
Coffee	2.81	3.02	0.21	+7.5*	3.01	3.06
Tea	3.38	3.62	0.24	+7.1*	4.06	2.32

The * symbol means that this result is significant.

### Meat, dairy products, and plant-based alternatives

A significant decrease in the frequency of consumption of milk and dairy products was observed when comparing ZR2 and ZR1 (2.99 d/wk at ZR2 vs. 3.4 d/wk at ZR1); at the same time, the frequency of consumption of plant-based milk alternatives increased from 2.18 d/wk at ZR1 to 2.76 d/wk at ZR2 (Table 7). Less meat and fewer sausages were also eaten (1.58 d/wk at ZR2 vs. 2.03 d/wk at ZR1) in favor of a significantly increased consumption of plant-based meat alternatives (1.22 d/wk at ZR2 vs. 0.85 d/wk at ZR1).

### Drinks

**FIGURE 6:**
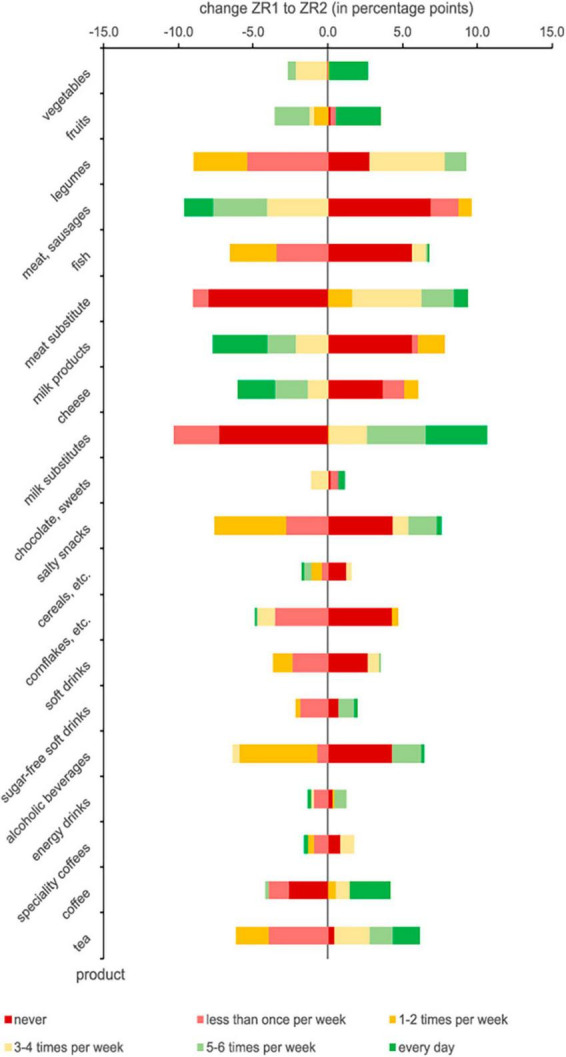
Changes in periodicity of food consumption from ZR1 to ZR2.

Significant increases in the frequency of consumption from ZR1 to ZR2 were found for coffee (2.81 d/wk at ZR1 to 3.02 d/wk at ZR2) and tea (3.38 d/wk at ZR1 to 3.62 d/wk at ZR2) (Table 7). The proportion of students who drank alcoholic beverages between 1 and 4 days per week at ZR1 decreased at ZR2. At the same time, there was an increase in the number of students consuming alcohol every day (4.1% at ZR2 vs. 1.9% at ZR1) and never (29.8% at ZR2 vs. 25.5% at ZR1) (Table 7 and Figure 6).

Bars with negative values indicate a decrease in the number of participants choosing the related category of consumption frequency. The wider the range, the greater the changes.

### Sustainability-related aspects

**FIGURE 7:**
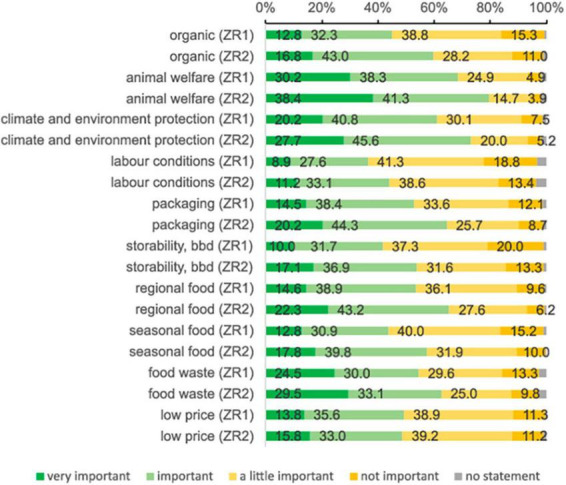
Relevance of sustainability-related food criteria during food purchase at ZR1 and ZR2.

At ZR1, the most relevant sustainability criteria were animal welfare (30.2 + 38.3%) and climate and environment protection (20.2 + 40.8%) (Figure 7). The fewest number of respondents stated that they found labor conditions important (8.9 + 27.6%). During the pandemic, organic products (59.8% at ZR2 vs. 45.1% at ZR1), climate and environment protection (73.3% at ZR2 vs. 61% at ZR1), seasonality (57.9% at ZR2 vs. 43.7% at ZR1), and storability (54% at ZR2 vs. 41.7% at ZR1) became significantly more important. However, all aspects were rated more positively than at ZR1, except for the low price for food, where the consent decreased marginally from 49.3 to 48.8%. Female students rated all aspects at ZR1 and ZR2 as more important than male students did.

### University catering

Students reported having visited university canteens at ZR1 1.55 days per week; cafeterias were visited half as often (0.65 d/wk) and vending machines almost never (0.11 d/wk) (Table 8). The frequency of usage in all instances decreased dramatically at ZR2 (Table 8). During the pandemic, students especially missed contact with others in the canteens (46.8 + 27.5%) and the function of canteens as meeting points (38.8 + 32.1%) (Table 9).

**TABLE 8:** Frequency of use of university catering.

		Total (d/wk) *n* = 959	Female (d/wk) *n* = 705	Male (d/wk) *n* = 239	East-G (d/wk) *n* = 342	West-G (d/wk) *n* = 615
Canteen	ZR1	1.55	1.42	1.91	1.67	1.49
	ZR2	0.31	0.28	0.37	0.77	0.05
	Change	−1.24*	−1.14*	−1.54*	−0.9*	−1.44*
Cafeteria	ZR1	0.65	0.62	0.73	0.33	0.83
	ZR2	0.06	0.05	0.08	0.10	0.03
	Change	−0.59*	−0.57*	−0.65*	−0.23*	−0.8*
Snack vending machine	ZR1	0.11	0.07	0.21	0.11	0.10
	ZR2	0.05	0.04	0.08	0.12	0.01
	Change	−0.06	−0.03	−0.13	0.01	−0.09
Coffee vending machine	ZR1	0.32	0.31	0.33	0.39	0.28
	ZR2	0.09	0.07	0.14	0.21	0.02
	Change	−0.23*	−0.24*	−0.19*	−0.18*	−0.26*

The * symbol means that this result is significant.

**TABLE 9:** Reasons for students to use the university catering and to miss it at ZR2.

Statement	Completely true (%)	True (%)	Not true (%)	Not true at all (%)	No statement (%)
Meeting point	38.8	32.1	7.7	7.4	14.0
Socializing	46.8	27.5	6.4	5.9	13.3
Social prices	17.5	27.7	19.8	14.4	20.5
Convenience	15.5	29.4	20.9	17.6	16.6
Versatility of food	11.8	21.6	22.6	24.0	20.0
Quality of food	6.0	19.5	28.1	24.0	22.4

## Discussion

The obtained sociodemographic data are congruent with the findings of previous studies, which enables a comparison and discussion of the data (8, 19, 41, 42). The four typical forms of housing for students were all reported at a similar rate. These results differ from previous data that identified shared flats as the dominant form of housing for students (11, 12). This observation is likely explained by the pandemic-associated lockdown, with students in shared flats moving back to their parents or to their partner (5, 7).

Male respondents stated significantly more often that they were not eating a healthy and balanced diet, as they also did in other studies. This phenomenon could be related to social expectations regarding beauty ideals, which affect mainly women (43, 44). Another explanation could be the previously mentioned characteristic that especially young females respond positively to health-associated DPs, including vegetarian and vegan diets (2, 35). The more frequent meat consumption of male students (2.6 d/wk at ZR2 vs. 1.2 d/wk at ZR1) matches this assumption. However, the percentage of vegetarian male students increased faster during ZR2 than the percentage of female students did.

Almost all students who started to follow a vegan diet at ZR2 had already been vegetarian for a certain amount of time before the lockdown. Most other students who began to follow a special diet at ZR2 did not follow any diet before and moved toward vegetarianism. These results confirm the findings of other studies (13, 15, 41). Especially students in the first three semesters, who had recently gained knowledge about healthy diets and nutrition in their lectures and had new social contacts, started to follow a vegetarian or vegan diet (Table 5). Hence, changes in their DPs could be attributed to influences from the study and other students (4). The more than 10% total increase in “non-meat-eating students” during the lockdown was higher than was found in the cross section of the population but was lower than the increase of 21% found in a study at the HAW Hamburg (45).

Social media has become highly relevant for young people, which is reflected in the importance of social media in gathering inspiration for their own nutrition and DPs. Especially during the COVID-19 pandemic, it was easier for students to use the internet than to meet friends or family members (7). Inspirational sources such as books, magazines, and television shows, which are more important for older people, were not important for the younger population (46). The most common argument is that “self-motivation” is a subjective term that cannot be addressed and discussed objectively. However, it is a statement of felt independence, self-control, or freedom to act (47), representing the positive thinking of students during the lockdown.

The 39.5% increase in the number of students who tried new recipes at ZR2 is comparable to the 55% increase in another sample in the study by Bühlmeier et al. (8). Generally, the frequency of fresh food preparation due to the SARS-CoV-2 pandemic has also been congruently described in other studies (19, 24, 48–50). The “Fachkraft 2030” study found that 83% of the surveyed students cooked fresh foods several times per week already before the pandemic, but 18% reported eating ready-to-eat products regularly, 43% regularly ate out of home, and only 3% frequently used delivery services (42, 51). The increasing trend of fresh cooking was already present before the pandemic but increased during the lockdown, especially for young people (8, 14), meaning that students react more or faster to extraneous influences than the general population (15, 19). However, only 22.8% of the students stated that they prepared fresh meals every day, which was less than half the proportion in the German population (14).

Countries and people with a low household income seem to skip meals more often and consume less fresh food than those with a high HNI (52). Moreover, increasing food insecurity has been reported (22, 53). This behavior could not be observed for students in our study, even though many of them had a low HNI. Fresh preparation of meals was chosen more often at ZR2 than at ZR1, and ready-to-prepare products were consumed less frequently (Table 6). The increased use of delivery services is contrary to the results of some other countries (54–56) but congruent with other findings Chen et al. (50) and the German population (41).

The increase in consumption of fruits and vegetables has been described before (8, 15, 53, 56–59). In Germany, the proportion of people eating vegetables or fruits every day increased insignificantly during the pandemic, from 70 to 72% (13, 14). Compared to Bühlmeier et al. (8) or Busch et al. (19) findings, where more than 20% of the respondents reported eating more fruits and vegetables, the students in the present sample ate only 2.6% more vegetables and 3% more fruits.

What is remarkable is the significantly increased consumption of legumes during the pandemic, which is also consistent with the results of the study by Maharat et al. (56). At ZR2, the proportion of students who stated they would never eat legumes increased. This increase can be explained by the fact that legumes are often used in university catering or restaurants (22, 60, 61). As the canteens closed, many students stopped consuming legumes. Legumes are an indicator of sustainability-oriented DPs, especially for vegetarians and vegans (62). The significant 7.8% increase in the frequency of consumption of legumes during the lockdown could suggest that the increased consumption of legumes may have occurred primarily among those who already consumed legumes in ZR1.

Influences of the pandemic on the sustainability-related aspects of students’ DPs could be linked to the consumption frequency of animal products and their plant-based alternatives. The decreasing frequency of consumption of meat, milk, and dairy products at ZR2, together with the significant increase in the consumption of alternatives, is evidence of changing DPs. A reduced consumption of dairy and meat products has also been shown in other studies with young adults and students, respectively (56, 63). It especially represents the sustainability-oriented behavior of the German society (38). At least 47% of the German population bought plant-based alternatives at least once (14). The students in this study, however, also consumed less cheese at ZR2, which is contrary to the general German population (62, 64). The reduced intake of milk or yogurt was compensated for by an increased consumption of cheese, but students in the present study did not seem to follow this trend. Even though there were almost no adequate plant-based cheese alternatives available at the beginning of the pandemic. Students substituting animal products with alternatives was special and likely originated from the high interest in sustainability-related topics (36, 62).

Even though the frequency of alcohol consumption did not change from ZR1 to ZR2, a characteristic change in the consumption pattern was observed: During the lockdown, students did not attend parties but started to drink more at home. Our results do not match those of previous studies (26, 48). The study results on alcohol consumption by students during the pandemic vary internationally. A measured increase in alcohol consumption was found in a survey on mental wellbeing during the pandemic (65). Another study states that alcohol consumption among students has fallen as they have moved back into their parents’ home during the lockdown and this may be a reason for a reduction in alcohol consumption (66). However, some surveys documented fewer purchases of alcohol during the pandemic, which can result in less frequent consumption (58).

Several studies have described and discussed an increased consumption of sweets during the pandemic (8, 57, 67–69). However, our data do not confirm these findings. Instead, a negligible decrease of 0.01 days per week was observed (Table 9). In a different study with students, a slight decrease was also observed (70). Since sweets and ready-to-eat products bear the distinct societal stigma of being “unhealthy,” undesirable foods, self-reporting of the consumption rates of these products is potentially delicate in terms of underreporting (62). Regarding these foods, men react differently than women, who are often suppressed by societal pressure to be slim (43). Hence, women react more sensitively to questions about sweets and might have overreported their consumption of sweets, while men tend to overreport ready-to-eat products, as they are a status symbol (44). Male students stated that they ate significantly more ready-to-eat products than did women (25.3 vs. 12.8%) and used food delivery services more frequently (4.9 vs. 2.1%).

Increased sensitivity to effects of private food consumption has been the subject of several studies (8, 19, 25, 49). Especially the importance of regionality and animal welfare matches the general trend described for Germany (13, 14). The significant increase in the consumption of organic products is notable, although it is associated with increased expenses (71). This result is therefore a strong signal for changed attitudes.

In addition to the established criteria, aspects such as packaging and storability have been surveyed since their importance increased not only for students, but also for other groups (14, 19, 41). Storability has become an indicator of the fear of food shortages (24). Regarding social and ethical motives in the present study, sensitivity toward animal welfare also outweighed the aspect of fair labor conditions in the food production and processing sector. In previous surveys, labor conditions also had the smallest consent of all sustainability-related criteria (72). However, it seems unlikely that students do not find labor conditions unimportant. This observation may be explained by a lack of information (19, 72).

Price consciousness, which had already decreased for several years before the pandemic (13, 14), was confirmed by our results. In contrast to findings by Busch et al. (19), in the present study, students seemed to prioritize the quality of food and to appreciate sustainability-related aspects even more at ZR2 than at ZR1. In this respect, the lockdown seemed to have sharpened their awareness of sustainability and health-oriented DPs.

The closure of university catering had a direct impact on students’ social interactions. They compensated for the lack of meals in canteens and cafeterias by preparing meals themselves and using takeaway and delivery services. Evidence of the shift toward sustainability-oriented diets was found in the increase in the number of vegetarian and vegan students, the consumption frequency of legumes, and the substitution of animal products with plant-based alternatives. Hence, the closure of university catering and the lockdown due to the COVID-19 pandemic had a substantial impact on the DPs of university students.

In conclusion, it can be stated that the results could give answers to the three underlying questions. The survey results show that the DPs of university students in Germany changed significantly during the SARS-CoV-2 pandemic. The increase in the consumption of legumes and plant-based alternatives indicate changes in DPs, led by sustainability-oriented criteria. Differences in these students compared with the general population became evident regarding the consumption of legumes, plant-based alternatives, snacks, sweets, and alcoholic beverages. The high interest in sustainable food and animal welfare confirms the results of previous studies and underline the relevance of these aspects for students. Despite dramatic changes in their daily routines, students seemed to adapt to more positive DPs than unfavorable ones, especially regarding the consumption of sweets and alcohol. Thus, their own motivation to eat more sustainably and healthily seems to have outweighed the negative emotions caused by a lack of social contact, a lower income, and the loss of everyday campus life. This makes it clear to how the DPs have changed and what the main motivations for this have been. The closed university catering also had an impact here. At the same time, the social component of these places came into effect as well.

## Data availability statement

The raw data supporting the conclusions of this article will be made available by the authors, without undue reservation.

## Ethics statement

Ethical approval was not required for the studies involving humans because participation in the study was anonymous. The studies were conducted in accordance with the local legislation and institutional requirements. The participants provided their informed consent to participate in this study by clicking the “continue” button in the start page.

## Author contributions

JD: Conceptualization, Investigation, Methodology, Project administration, Writing – review and editing, Writing – original draft. AB: Writing – original draft, Formal Analysis, Visualization. SL: Conceptualization, Methodology, Supervision, Writing – original draft. CS: Supervision, Writing – review and editing. MS: Writing – original draft, Conceptualization, Methodology, Supervision, Writing – review and editing.
